# Book Reviews

**Published:** 1800-02

**Authors:** 


### CHEMISTRY.

*Handbuch der theoretischen und praktischen Chemie,* i. e. A Manual of the Theory and Practice of Chemistry, by J. F. A. GÖTTLING, Professor in the University of Jena, and Member of several learned societies. Part I. containing the theory, 1798. Part II. containing the practice, 1799; together 1089 pp. 8vo. (Price 3 rixdollars 4 gr. or about 13 shillings.) *Jena,* in the Academical Shop.

THIS new work of M. Göttling contains the principles of a science which the celebrated author has long and successfully cultivated; he has enriched it with many important observations and experiments, as well as every new fact and discovery which he had an opportunity of demonstrating in his lectures to an audience that, in point of number and respectability, has been equal to that of the celebrated Dr. Black, at Edinburgh.

The plan adopted by the author, as well as his method of treating the different objects of chemistry, together with his endeavours to render this a complete elementary work of that useful science, deserve every commendation. As our limits do not permit us to give an analytical view of this work, we shall content ourselves with making a few general observations on the theory peculiar to Mr. Göttling. From the latest experiments he appears to be convinced, that the purest oxygen gas, on being treated with phosphorus at a low temperature, is changed into azotic gas; that consequently the latter species of air contains the same ponderable basis as the former; and that azote, though its existence has been admitted in other combinations, upon the authority of Lavoisier, neither exists here nor in azotic gas. Caloric is by him considered as a fluid which so readily penetrates all bodies, that for this very reason it is not ponderable by any apparatus which could possibly be invented; and though he does not enter into an investigation of the materiality or immateriality of this substance, or the cause of heat, yet it is pretty evident from his details, that he supposes it to approach to something of a material nature; for the properties which he attributes to it, can be predicated only of matter. This substance appears in other respects to be intimately connected with the cause of light, and in this combination forms fire; while its elastic property seems to be considerably raised or increased by light; and in this association, together with oxygen, it forms the nitric acid. But whether light and heat are the same substance, and whether their different effects depend upon certain undetermined modifications, or whether that which is called light, in its combination with the bodies it meets with on its passage, is changed into what we term heat, on these questions we cannot decide with any degree of certainty at present: yet, we are less liable to be involved in hypotheses, if we carefully separate the phænomena resulting from fire, and admit light and heat as two simple substances distinct from each other. Light is, according to him, him, a conftituent part of all inflammable bodies, and neither fulphur, nor phofphorus, the metals, &c. make any exception from this general rule; for the firft mentioned fubftance is compofed of the bafe of fulphur and the caufe of light, phofphorus of its own bafe and a fimilar caufe, and the metals cannot be otherwife accounted for, becaufe in them the caufe of light is combined with peculiar metallic bafes. Hence, the bafe of fulphur forms combinations of a particular kind with the bafes of metals; and fuch were the productions obtained by the Dutch chemifts, who, in their experiments upon the difengagement of light, could not effect the combuftion of fulphur, though they heated that fubftance with metals, in various fpecies of factitious airs.

[To be continued in our next Number.]

---

*A General View of the Nature and Objects of Chemiftry, and its Application to Arts and Manufactures*; by William Henry, Member of the Royal Medical and Natural Hiftory Societies of Edinburgh, &c. 8vo. pp. 44. 1s. 1799. Johnfon.

THIS interefting pamphlet contains the fubftance of an introductory addrefs to a courfe of chemical lectures delivered by the author, at Manchefter, during the laft winter. As Mr. Henry propofes to repeat thefe lectures, he was induced to publifh their objects and plan; efpecially as, in his opinion, this fketch affords a more ample detail than has, perhaps, been hitherto given of the *general ufes and applications of chemiftry.*

Inftead of filling thefe pages with the barren hiftory of chemical fcience, and amufing the reader with the biography of thofe deluded mortals who feem to have vied with each other in wafting their time in idle fpeculations, and the abfurd purfuits of alchemy and aftrology, the author has, in thefe few fheets, fubftituted a brief view of the nature and objects of chemiftry, of its connection with the arts and other fciences, and an outline of the plan on which the following Lectures will be conducted.

" Natural Philofophy," fays he " in its moft extenfive fenfe, is a term comprehending every fcience that has for its objects the properties and affections of matter. But it has attained, by the fanction of common language, a more limited fignification; and chemiftry, though ftrictly a branch of Natural Philofophy, is generally regarded as a diftinct fcience. Between the two, it may perhaps be difficult to mark out precifely the line of feparation; but, an obvious character of the facts of Natural Philofophy, is, that they are always attended with fenfible motion; and the determination of the laws of motion is peculiarly the office of its cultivators. Chemical changes, on the other hand, of the moft important kind, often take place without any apparent motion, either of the mafs, or of its minute parts; and where the eye is unable to perceive that any change has occurred. The laws of gravitation, of central forces, and all the other powers that fall under the cognizance of the natural philofopher, produce, at moft, only a change a change of place in the bodies that are influenced by them. But, in chemical changes, we may always obferve an important difference in the outward properties of things. Two highly corrofive fubftances, by uniting chemically together, may become mild and harmlefs; the combination of two colourlefs fubftances may prefent us with a compound of brilliant complexion, and the union of two fluids with a compact and folid mafs.

"Chemiftry, therefore, may be defined that fcience, whofe object is to difcover and explain the changes of compofition, that occur among the integrant and conftituent parts of bodies.

"From this definition, it may readily be conceived how wide is the range of chemical inquiry; and by applying it to the various events that daily occur in the order of Nature, we fhall be enabled to feparate them with accuracy, and to allot to the fciences of Natural Philofophy and Chemiftry, the proper objects of the cultivation of each. Whenever a change of place is a neceffary part of any event, we fhall call in the aid of the former. When this condition may be difpenfed with, we fhall refort to Chemiftry for the light of its principles; but it will be often found, that the concurrence of the two fciences is effential to the full explanation of phænomena. The water of the ocean, for example, is raifed into the atmofphere by its chemical combination with the matter of heat; but the clouds that are thus formed, maintain their elevated fituation, by virtue of a fpecific gravity, lefs than that of the lower regions of the air; a law, whofe difcovery and application are due to the natural philofopher ftrictly fo called."

